# Supplementary material for: Transcriptome Analysis Provides Insights into the Mechanism of the Transcription Factor AaCrz1 Regulating the Infection Structure Formation of Alternaria alternata Induced by Pear Peel Wax Signal
Source: Int J Mol Sci. 2024 Nov 7;25(22):11950. doi: 10.3390/ijms252211950 (PMC11593592; doi:10.3390/ijms252211950)
Supplement: Supplementary file 1 [file ijms-25-11950-s001.zip › ijms-3232525-Supplementary Material.pdf]

Supplementary Table S1

DEGs encoding proteins in three selected pathways.

Gene ID	Symbol	Annotation	log2FC
**ko00500 Starch and sucrose metabolism**			
ncbi_29119758	*gyg-1*	GYG1, GYG2; nucleotide-diphospho-sugar transferase	-1.11659648
ncbi_29110990	*amyS*	AMY, amyA, malS; alpha-amylase	-2.19313077
ncbi_29121270	*mal1*	IMA, malL; oligo-1,6-glucosidase	-1.24398782
ncbi_29120998	*bglF*	beta-glucosidase	-1.22867844
ncbi_29119646	*treh*	*TREH*, treA, treF; alpha,alpha-trehalase	-1.73556472
ncbi_29119076	*GDB1*	AGL; glycogen debranching enzyme	-1.33661934
ncbi_29108996	*EXG1*	glucan 1,3-beta-glucosidase	-1.20181946
ncbi_29109130	*OTS1*	TSL1, TPS3; trehalose 6-phosphate synthase complex regulatory subunit	-1.21608064
ncbi_29116806	*celb*	endoglucanase	-1.94209916
**ko04136 Autophagy-other**			
ncbi_29109885	*ATG4*	cysteine protease *ATG4*	1.401838573
ncbi_29111274	*ATG9*	autophagy-related protein 9	1.094709678
ncbi_29117977	*ATG1*	MAP/microtubule affinity-regulating kinase 4	1.641443
**Ko02010 ABC transporters**			
ncbi_29117678	*atrA*	ATP-binding cassette, subfamily G	-1.05314506
ncbi_29110931	*atrB*	ATP-binding cassette, subfamily G	-1.26417730

Supplementary Table S2

DEGs regulated by *AaCrz1*.

Classification	Gene ID	Symbol	Annotation	log2FC
**Cell wall degradation enzyme**				
	ncbi_29117201	*cutA*	cutinase	-1.34495570
	ncbi_29116806	*celb*	endoglucanase	-1.942099169
	ncbi_29120322	eglD	endoglucanase-like protein II	-1.3548518557
	ncbi_29115410		endoglucanase-5	-5.16992500
	ncbi_29111957	*celb*	Endoglucanase B	-1.526629190
**ROS**				
	ncbi_29111723	*sod-2*	superoxide dismutase mitochondrial precursor	3.248560216
	ncbi_29109411	*cat-1*	katE, CAT, catalase-domain-containing protein	-1.13282728
	ncbi_29114347	*catB*	catalase-domain-containing protein	-1.18028326
	ncbi_29121404	*CAT1*	catalase-domain-containing protein	-4.55927445
	ncbi_29116487	*Erg3*	ERG4/ERG24 ergosterol biosynthesis protein	-1.777186585
**ion transport**				
	ncbi_29117801	*pmc1*	plasma membrane calcium-transporting ATPase 2	-6.73019016
	ncbi_29118340	*VCX1*	vacuolar calcium ion transporter /H(+) exchanger	-3.5703011
	ncbi_29119593	*VCX1*	vacuolar calcium ion transporter /H(+) exchanger	-1.1858913
	ncbi_29120094	*pmc1*	plasma membrane calcium-transporting ATPase 3	-3.9910511
	ncbi_29109809	*RCAN2*	Calcipressin-domain-containing protein	-4.215547004

Supplementary Table S3

Identification of proteins interacted with AaCrz1.

motif_id	motif_alt_id	target_gene_id	target_gene_symbol	matched_sequence
MA0285.1	CRZ1	ncbi_29111171	cysD	CTCAGCCAC
MA0285.1	CRZ1	ncbi_29115175	tor2	CTCAGCCAC
MA0285.1	CRZ1	ncbi_29119021	fer6	CCAAGCCAC
MA0285.1	CRZ1	ncbi_29121704	chs-1	CTCAGCCAC
MA0285.1	CRZ1	ncbi_29117972	pho-5	CTAAGCCAC
MA0285.1	CRZ1	ncbi_29115620	VCX1	CTCAGCCAC
MA0285.1	CRZ1	ncbi_29109761	csbC	CAACGCCAC
MA0285.1	CRZ1	ncbi_29109602	adh1	CTCAGCCAC
MA0285.1	CRZ1	ncbi_29111454	SPAC26F1.07	CTAAGCCAC
MA0285.1	CRZ1	ncbi_29119703	ght6	CACAGCCAC
MA0285.1	CRZ1	ncbi_29116084	CTHT_0055700	CCAAGCCCC
MA0285.1	CRZ1	ncbi_29109810	RPS6KA2	CTCAGCCTC
MA0285.1	CRZ1	ncbi_29112137	RVS167	CACCGCCAC
MA0285.1	CRZ1	ncbi_29112337	RSR1	CACCGCCAC
MA0285.1	CRZ1	ncbi_29113467	ATP1A1	CACAGCCAC
MA0285.1	CRZ1	ncbi_29119518	ADH7	CCCAGCCAC
MA0285.1	CRZ1	ncbi_29114118	NHA1	CCCAGCCAC
MA0285.1	CRZ1	ncbi_29114556	srk1	CTCAGCCTC
MA0285.1	CRZ1	ncbi_29117115	gld2	CTACGCCAC
MA0285.1	CRZ1	ncbi_29117335	gar1	CTCAGCCAC
MA0285.1	CRZ1	ncbi_29117441	PTPRT	CTCAGCCAC
MA0285.1	CRZ1	ncbi_29115069	atrA	CTCAGCCAC
MA0285.1	CRZ1	ncbi_29117801	pmc1	CAAAGCCTC
MA0285.1	CRZ1	ncbi_29115620	VCX1	CTCAGCCAC
MA0285.1	CRZ1	ncbi_29118482	Map3k6	CTCAGCCAC
MA0285.1	CRZ1	ncbi_29119518	ADH6	CCCAGCCAC
MA0285.1	CRZ1	ncbi_29118340	VCX1	CTCCGCCAC
MA0285.1	CRZ1	ncbi_29120094	pmc1	CTCAGCCTC
MA0285.1	CRZ1	ncbi_29120897	GCY1	CAAAGCCAC
MA0285.1	CRZ1	ncbi_29120173	CAT1	CAGAGCCAC

Supplementary Table S4

Primers used in this study

Gene	Primer sequences (5' - 3')
Aacelb-F	ATACCGCCTACGATGTAACCA
Aacelb-R	TTGAGATACGCCATATTGCTG
AaccutA-F	ATGCCGACCGTGACGAGACC
AaccutA-R	GAAAGCCGCAGCCGTAGGAG
AabglF-F	GTGGCACAACGGTGACAAAC
AabglF-R	GGCAAGTGAGCGAACAGGAC
AaEXG1-F	CGGCTCTGTTGTCATTATCG
AaEXG1-R	AGGCTTACTGGTCCTTGTCC
AaatrA-F	CTGGTTACAGCAAGGGTTCA
AaatrA-R	GTAGACGACATCACGCCAAG
AaatrB-F	ACCCATGACAGAAGACGATC
AaatrB-R	TTAGTGTTAGACTTGCTGAGGC
AaSOD-F	GAGCAAAGGCTGTCTATCGTG
AaSOD-R	CCTTGCCGTTCTGGTATTGG
AaCAT-F	CCATCAACCCACCTCACTCA
AaCAT-R	TGCGGTAACCATCAGGAATA
AaErg3-F	CGGCAGCACGACTGTAGTTT
AaErg3-R	GGTCAAAGGCTCAGGTGTCTC
AaATG4-F	GGTCAAAGGCTCAGGTGTCTC
AaATG4-R	TCGTAGTCTTCCTGCGATGTTAT

AaATG9-F	GAGGAACCAGGCAATAACCG
AaATG9-R	TAGACACCAACACCAGCACC
AaVCX1-F	CTCAAGCAGGGCGAAATCAG
AaVCX1-R	CAGCATCCGAGCACGAGAAG
Aapmc1-F	CGAGATTGAGGCTACACCACT
Aapmc1-R	CCACCAACAAAGCGAAACAA
AaRCAN2-F	ATCTTCCAACTATCAACCCTTACC
AaRCAN2-R	TTGTATCGCAGAGTCAATGTCG

Supplementary Table S5

PCR reaction system

Name	Dosage (μL)
2×SYBR® Green Pro Taq HS Premix	10
forward primer (10 μM)	1
reverse primer (10 μM)	1
cDNA	1.5
ddH_2O	6.5

Supplementary Table S6

PCR conditions

Temperature (℃)	Time (s)	Cycle
95	30	1
95	5	40
60	30	40
